# Diagnostic yield and clinical utility of whole exome sequencing using an automated variant prioritization system, EVIDENCE

**DOI:** 10.1111/cge.13848

**Published:** 2020-09-17

**Authors:** Go Hun Seo, Taeho Kim, In Hee Choi, Jung‐young Park, Jungsul Lee, Sehwan Kim, Dhong‐gun Won, Arum Oh, Yena Lee, Jeongmin Choi, Hajeong Lee, Hee Gyung Kang, Hee Yeon Cho, Min Hyun Cho, Yoon Jeon Kim, Young Hee Yoon, Baik‐Lin Eun, Robert J. Desnick, Changwon Keum, Beom Hee Lee

**Affiliations:** ^1^ Division of Medical genetics 3billion Inc. Seoul South Korea; ^2^ Biomedical Research Center, Asan Institute for Life Sciences University of Ulsan College of Medicine Seoul South Korea; ^3^ Medical Genetics Center, Asan Medical Center University of Ulsan College of Medicine Seoul South Korea; ^4^ Department of Pediatrics, Asan Medical Center University of Ulsan College of Medicine Seoul South Korea; ^5^ Department of Internal Medicine Seoul National University Hospital, Seoul National University College of Medicine Seoul South Korea; ^6^ Department of Pediatrics Seoul National University Hospital, Seoul National University College of Medicine Seoul South Korea; ^7^ Department of Pediatrics, Samsung Medical Center Sungkyunkwan University School of Medicine Seoul South Korea; ^8^ Department of Pediatrics, School of Medicine Kyungpook National University Daegu South Korea; ^9^ Department of Ophthalmology, Asan Medical Center University of Ulsan Seoul South Korea; ^10^ Department of Pediatrics Korea University College of Medicine Seoul South Korea; ^11^ Department of Genetics and Genomic Sciences, Icahn School of Medicine Mount Sinai Medical Center New York New York USA

**Keywords:** automated prioritization system, genetic diagnosis, variant, whole exome sequencing

## Abstract

EVIDENCE, an automated variant prioritization system, has been developed to facilitate whole exome sequencing analyses. This study investigated the diagnostic yield of EVIDENCE in patients with suspected genetic disorders. DNA from 330 probands (age range, 0‐68 years) with suspected genetic disorders were subjected to whole exome sequencing. Candidate variants were identified by EVIDENCE and confirmed by testing family members and/or clinical reassessments. EVIDENCE reported a total 228 variants in 200 (60.6%) of the 330 probands. The average number of organs involved per patient was 4.5 ± 5.0. After clinical reassessment and/or family member testing, 167 variants were identified in 141 probands (42.7%), including 105 novel variants. These variants were confirmed as being responsible for 121 genetic disorders. A total of 103 (61.7%) of the 167 variants in 95 patients were classified as pathogenic or probably to be pathogenic before, and 161 (96.4%) variants in 137 patients (41.5%) after, clinical assessment and/or family member testing. Factor associated with a variant being regarded as causative includes similar symptom scores of a gene variant to the phenotype of the patient. This new, automated variant interpretation system facilitated the diagnosis of various genetic diseases with a 42.7% diagnostic yield.

## Graphical abstract

Schematic diagram showing the number of patients with and without variant identification and family member testing and the proportion of variant classification.

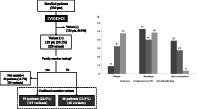

## 1 INTRODUCTION

To date, of the over 7000 mendelian disorders, more than 5000 have been shown to result from defects in a specific gene; pathogenic gene mutations for the rest continue to be discovered, primarily by whole exome sequencing (WES).^1^, ^2^

In a group of patients suspected to have genetic diseases, the diagnostic rate of WES has been found to range from 30% to 40%, a variation that may be attributed to the numbers and phenotypes of enrolled patients and the anthropologic characteristics of study cohorts.^3^, ^4^, ^5^, ^6^, ^7^, ^8^, ^9^, ^10^

Whole genome studies, such as, WES, are time‐consuming and labor‐intensive, requiring clinical geneticists, and bioinformaticians to match large numbers of candidate variants with various clinical symptoms in each subject analyzed.^11^ Moreover, in the absence of supporting data, many variants remain variants of uncertain significance (VUS), limiting the ability to confirm genetic diagnoses.^12^

Guidelines of the American College of Medical Genetics and Genomics (ACMG) attempted to prioritize genetic variants, which led to the development of several bioinformatic tools.^13^, ^14^ These tools; however, have limited ability to accurately predict the pathogenicity of each variant. Phenotype‐centric interpretation methods developed using several computational tools, which automatically prioritized the genetic variants in each patient and ranked them according to the biological function of each gene; the molecular, structural, and charge impact of the variant; and the relationship between the variant gene and the phenotype of the patient.^9^, ^15^, ^16^ Although these approaches noticeably reduced the number of candidate variants responsible for the disease phenotype in each patient, the numbers varied among studies, without significantly improving genetic diagnostic rates, which have remained at approximately 30% to 35%.^5^, ^16^

This study describes a new, streamlined, automated variant prioritization system, termed EVIDENCE (3billion Inc., Seoul, South Korea), which analyses over 100 000 variants, according to ACMG guidelines,^17^ and prioritizes variants based on each phenotype of each patient within minutes. A symptom suggestion system based on Human Phenotype Ontology (HPO) was created to capture most patient phenotypes. Finally, the EVIDENCE system was able to calculate “similarity scores” between the clinical phenotypes suggested by the candidate variants and actual patient phenotypes, to match this score with the genetic diseases listed in the OMIM database (www.omim.org). This pilot study found that EVIDENCE significantly improved the speed and rate of diagnoses of a variety of genetic diseases.

## 2 MATERIALS AND METHODS

### 2.1 Recruitment of patients

The study enrolled an unselected series of 330 consecutive patients, clinically suspected of carrying a genetic disorder, from 330 non‐consanguineous families, who presented at the Medical Genetics Center, Asan Medical Center, Seoul, South Korea, from April 2018 to August 2019. Their detailed demographic and clinical characteristics, including age and diagnosis at presentation, sex, family history, laboratory findings, radiologic findings, and genetic testing results, were reviewed.

Patients aged ≥5 months were included if they were strongly suspected by clinicians of having a genetic disease and were undiagnosed, despite application of conventional genetic tests, such as, chromosome analyses, chromosome microarray, or single or targeted gene panel testing. Patients aged <5 months were included if they had a congenital anomaly in one or more major organs, including the brain or the heart; or the gastrointestinal, urological, or musculoskeletal systems; or if they were strongly suspected by clinicians or radiologists of having a genetic disease.

Informed consent was obtained from patients or their legal guardians after genetic counseling regarding the WES test. The study was approved by the Institutional Review Board for Human Research of the Asan Medical Center (IRB numbers: 2018‐0574 and 2018‐0180).

### 2.2 Whole exome sequencing, variant calling, and variant annotation

Blood, saliva, or buccal swab samples were collected from each patient, and genomic DNA was extracted from each sample. All exon regions of all human genes (~22 000) were captured using the Agilent SureSelect kits (version C2, December 2018) and sequenced using the NovaSeq platform (Illumina, San Diego, CA). The quality of FASTQ files obtained by sequencing with the Illumina Novaseq 6000 was assessed using FASTQC (http://www.bioinformatics.babraham.ac.uk/projects/fastqc/). Subsequently, the base and sequence adapters with low base quality were removed using Trimmomatic.^18^ Pre‐processed FASTQ files were aligned to the reference sequence (original GRCh37 from NCBI, February 2009) by BWA‐MEM (v.0.7.17).^19^ Aligned BAM files were sorted and extracted using the statistical metric by samtools (v.1.9).^20^ Duplication was marked by Picard (v.2.20.8) (http://broadinstitute.github.io/picard/). Single nucleotide variants and indel variants were called by HaplotypeCaller of GATK (v.3.8).^21^ Finally, variant call formats (VCF) were generated. The mean depth of coverage was 100 X (>10 X = 99·2%).

### 2.3 EVIDENCE: Prioritization of variants and symptom suggestion system

The streamlined variant prioritization software program, EVIDENCE, was developed in‐house to prioritize variants based on ACMG guideline and the phenotype of each patient and to interpret these variants accurately and consistently. This system has three major steps: variant filtration, classification, and similarity scoring for patient phenotype. In the first step, allele frequency was estimated in population genome databases, including gnomAD (http://gnomad.broadinstitute.org/) and 3billion Inc. (https://3billion.io/).^22^ Common variants with a minor allele frequency of >5% were filtered out in accordance with rule BA1 of the ACMG guidelines.^17^

In the second step, Evidence of the pathogenicity of the variants was obtained from disease databases, including OMIM (www.omim.org), ClinVar, and UniProt; the factors included gene function, domain of interest, mechanism of development, inheritance pattern, and clinical relevance of the disease.^1^, ^23^, ^24^ The predicted functional or splicing effect of each variant and its degree of evolutionary conservation was evaluated using several in silico tools, including REVEL, adaptive boosting, and random forest score.^25^, ^26^ Scores above 0.5 in each tool predicted a detrimental effect on the variant. The pathogenicity of each variant was evaluated according to the recommendations of the ACMG guidelines.^17^ In the third step, the clinical phenotype of each proband was transformed to its corresponding standardized HPO term and was assessed to measure the similarity with each of ~7000 rare genetic diseases.^27^, ^28^ The similarity score between the phenotype of each patient and symptoms associated with that disease, caused by prioritized variants, according to ACMG guidelines, ranged from 0 to 10. For any given symptom in a patient, the symptom was compared with each of the known symptoms of a target disease. At each comparison of two symptoms, the maximal depth of a common ancestor node of two symptoms was registered as a weight candidate. As the given symptom of the patient was compared with each of the disease symptoms, the number of weight candidates and the number of disease symptoms were the same. The weight of a symptom was set as the maximum value of the registered candidate weights. Weights were calculated for all patient symptoms and averaged to S1. Using the same procedure, all known symptoms of the target disease were weighted and averaged to S2. The value, (S1 + S2)/2, was used as the score for symptom similarity between the patient and the target disease. The formula used to calculate the similarity score is (supplementary file 1):

(1) $$w\left(p,S\right)=\max\left\{s\in S:\mathrm{MC}A_{d}\left(p,s\right)\right\}$$

(2) $$d\left(S_{p},S_{d}\right)=\frac{1}{|S_{p}|}\sum_{s\in S_{p}}w\left(p,S_{p}\right)$$

(3) $$\text{score}\left(S_{p},S_{d}\right)=\frac{1}{2}\left(d\left(S_{p},S_{d}\right)+d\left(S_{d},S_{p}\right)\right)$$

Incidental findings were not included in this study. Finally, EVIDENCE prioritized variants that were classified as pathogenic, probably pathogenic, or VUS according to ACMG guidelines, were categorized into a three tier system based on the Bayesian score.^29^ The first tier was scored above 0.9, the second tier above 0.499, and the third tier above 0.1. These variants were ranked higher as the similarity score within each tier was high. A diagram highlighting each step of the filtering process used for variants and databases related to this process are presented in Supplementary Figure 1 and Table S1, respectively.

The entire process of genetic diagnosis, including processing of raw genome data, determining variant prioritization, and measuring the similarity between each phenotype and disease, was integrated and automated into a computational framework.

### 2.4 Variant interpretation and confirmation

Relevant candidate variants, including VUS, based on EVIDENCE, were manually reviewed, related to applied ACMG rules and disease characteristics, and then selected by medical geneticists. After another examination in the outpatient clinic, the DNA of each patient and/or their parents was subjected to Sanger sequencing to confirm the candidate variant(s). If necessary, a chromosomal microarray was performed to assess uniparental disomy (UPD) or structural variants after Sanger sequencing of the family members.

### 2.5 Statistical analysis

All statistical analyses were performed using the R studio software (version 3.5.1). Principal component analysis (PCA) of symptoms and genetic variations required construction of a symptom matrix and a genetic variation matrix for each patient, with entries of 1 for patient *j* having symptoms or variant *i*, and entries of 0 otherwise. All pathogenic variants aggregated from the entire patient cohort were used. 10 types of functional variations were treated separately, resulting in 1285 combinations of genetic and functional variants. The entries in both matrices were calculated using a custom‐made program and an Eigen C++ linear algebra library, with *P* < .05 considered statistically significant.

## 3 RESULTS

### 3.1 Patient demographics

The demographic characteristics of the 330 patients have been shown in Table 1. Mean ages at clinical presentation and when WES was performed were 5.9 ± 12.9 years (range, 0‐68 years) and 11.9 ± 16.2 years (range, 0‐70 years), respectively. Of the 330 patients, 246 (74.5%) were under 18 years of age. Patients manifested a broad range of phenotypes across organ systems. The average number of systems manifesting phenotypic abnormalities per patient was 4.5 ± 5.0. Abnormalities in the nervous system were the most frequent, observed in 60% of patients, followed by those in the musculoskeletal system (53.9%), the head and neck (43.3%), the cardiovascular system (26.9%), and the endocrine system and metabolism (24.2%) (Table 1). Of the total 16 000 HPO terms, 550 terms were identified in 330 patients. These terms were broadly distributed throughout the genome, with the HPO terms matching patient symptoms highlighted in red, as visualized by the Cytoscape 3.7.1 (Supplementary Figure 2). These findings indicate that the phenotypes of the patients in this study represent almost the entire range of human disease phenotypes described to date.

**TABLE 1:** Demographic and clinical characteristics of the patient cohort

Category	Number (%)
Sex (male:female)	164:166 (49.7:50.3%)
Age at presentation, years	5.9 ± 12.9 (range, 0‐68)
Age at time of whole exome sequencing, years	11.9 ± 16.2 (range, 0–70)
Average number of overlapping phenotypes	4.3 ± 5.0
Nervous system, including behavior and/or cognition	198 (60%)
Head or neck, including facial dysmorphism	143 (43.3%)
Eye system	79 (23.9%)
Ear system	62 (18.8%)
Cardiovascular system	89 (26.9%)
Respiratory system	17 (5.2%)
Gastrointestinal system	56 (16.9%)
Genitourinary system	76 (23%)
Endocrine and metabolism/homeostasis system	80 (24.2%)
Musculoskeletal and limb system	178 (53.9%)
Connective tissue system	21 (6.4%)
Blood and immune system	57 (17.3%)
Skin	56 (16.9%)
Neoplasm	29 (8.8%)
Growth	74 (22.4%)
Abnormality of prenatal development or birth	42 (12.7%)
Previous genetic analysis	214 (64.8%)
Karyotype	123 (37.3%)
Fluorescence in situ hybridization	8 (2.4%)
Multiplex ligand dependent probe amplification	45 (13.6%)
Array comparative genome hybridization	6 (1.8%)
Single gene test	93 (28.2%)
Targeted exome sequencing or panel test	38 (11.5%)
Mitochondrial full genome sequencing	20 (6.1%)

*Note*: Results presented as mean ± SD or as number (%).

Of the 330 patients, 214 (64.8%) underwent genetic testing before WES. Further, 38 patients underwent targeted exome sequencing, which included 4813 OMIM genes, and 6 underwent array comparative genome hybridization, with none showing diagnostic variants. A total of 93 patients underwent single gene testing for monogenic disorders. Other genetic tests included karyotyping and/or fluorescence in situ hybridization (*N* = 131), multiplex ligation‐dependent probe amplification analyses for chromosomal microdeletion or duplication syndromes (*N* = 45), and mitochondrial full genome sequencing analysis (*N* = 20). No test revealed a specific diagnosis in the patients tested.

### 3.2 Diagnostic yield and classification of identified variants

**FIGURE 1:**
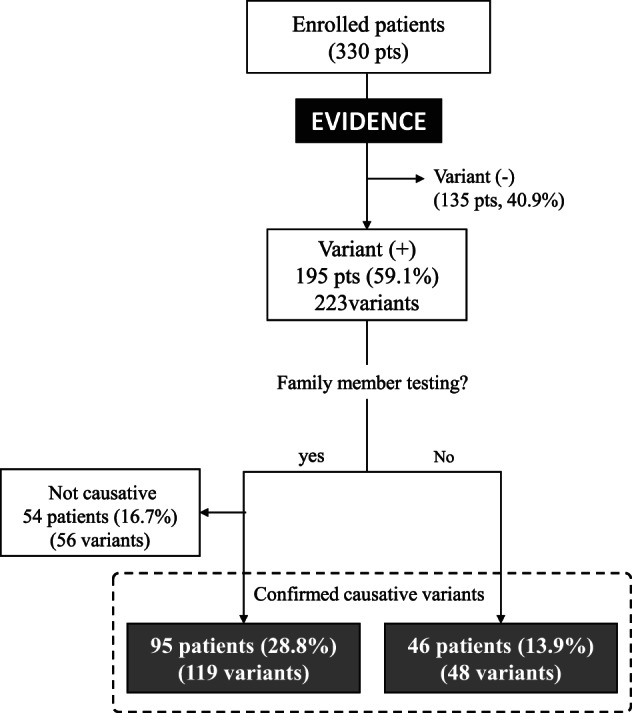
Schematic diagram showing the number of patients with and without variant identification and family member testing

The number of patients with variants and the identity of these variants have been summarized in Figure 1. EVIDENCE provided list of an average 65 variant‐disease pairs based on the ACMG guideline and similarity score. Medical geneticists and bioinformaticians evaluated each candidate variant‐disease pair and selected the variant‐disease most closely associated with the phenotype of each patient.

EVIDENCE identified 223 variants, including 121 VUS, in 195 (59.1%) of 330 patients. Among these, 175 variants from 149 (45.2%) patients were assessed by Sanger sequencing and family member testing, with 119 variants in 95 (28.8%) patients confirmed as being causative. In addition, 48 variants from 46 (13.9%) patients were confirmed, based on the function of the identified gene and the predicted pathogenicity of the variant, in addition to its frequency in the general population. In summary, 167 variants, including 105 novel variants, in 141 (42.7%) patients were confirmed as being responsible for 121 genetic disorders. The remaining 56 variants in 54 (16.3%) patients were not regarded as causative because they were inherited from an asymptomatic parent, and the putative gene represented a dominant disorder with expected high penetrance or was found in cis in a recessive disorder.

Rates of diagnosis did not differ significantly between patients who did and did not undergo genetic testing before WES (44.5% [93/209] vs 41.4% [48/116], *P* = .491).

The inheritance pattern of identified variants in the 141 patients was autosomal dominant (*N* = 99, 70.2%), autosomal recessive (*N* = 34, 24.1%), and X‐linked (*N* = 9, 6.4%). Of the 167 confirmed variants, 56 (33.5%) were confirmed as being de novo, and 23 (13.8%) were assumed to be de novo. Moreover, 49 variants from 25 patients, inherited in an autosomal recessive manner, were detected in a trans pattern.

**FIGURE 2:**
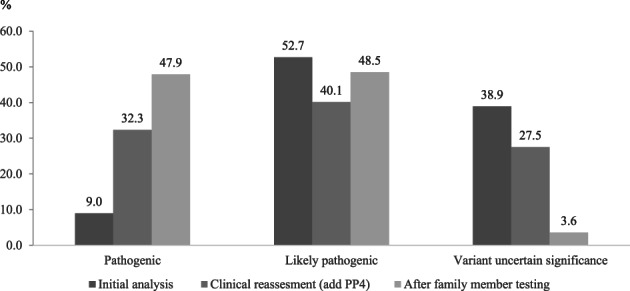
Distribution of the probably pathogenicity of identified variants by EVIDENCE before family member testing, after addition of phenotypic specific rules (PP4), and after family member testing

According to ACMG guidelines, 15 (9%) variants were classified as pathogenic, 88 (52.7%) probably pathogenic, and 65 (38.9%) of uncertain significance (Figure 2). After clinical assessment, including biochemical tests, imaging analysis, and physical examination, in the follow‐up with identified variants, 54 (32.3%) variants were classified as pathogenic, 67 (40.1%) probably pathogenic, and 46 (27.5%) of uncertain significance. After subsequent family member testing, 80 (47.9%) variants were classified as pathogenic, 81 (48.5%) probably pathogenic, and 6 (3.6%) of uncertain significance. In total, 137 patients (41.5%) had pathogenic or probably pathogenic variants. Details in all variants and diseases have been listed in Table S2.

With additional microarray, patients 182 and patient 241 had maternal UPD 14 and maternal UPD 9, respectively, showing a loss of heterozygosity encompassing the entire chromosome 14 and 9. Patient 182 and 241 had homozygous variant of c.713 C > T (p.Ser238Phe, probably pathogenic) in the *SLC7A7* gene and c.615 G > T (p.Met205Ile, VUS) in the *FRRSIL* gene, respectively. The family member testing detected the heterozygous variant only in the mother in both patients. We performed microarray because two patients showed highly specific phenotype associated with each variant.

### 3.3 Characteristics of confirmed variants

The characteristics of 167 variants, confirmed to be disease‐causing, and 56 variants, designated as non‐disease‐causing, were compared based on ACMG guidelines and symptom similarity. Of the 108 pathogenic or probably pathogenic variants, 103 (95.4%) were confirmed as being causative, in contrast to 69 (527.0%) of the 121 VUS. Of the 56 variants regarded as being non‐disease‐causing, 52 (92.8%) were classified as being of uncertain significance, whereas only 4 (7.1%) were classified as probably pathogenic; These four variants were found in the asymptomatic parents of a child with an autosomal dominant disorder with an expected high penetrance (Spastic Paraplegia, Intellectual Disability, Nystagmus, and Obesity, Sialuria, congenital muscular dystrophy, Neurodevelopmental Disorder with Dysmorphic Facies and Distal Limb Anomalies). Patient 91 with c.850del in the *KIDINS220* gene (NM_020738.2) showed failure to thrive, microcephaly, global developmental delay, mild dysmorphism, that is, not consistent with phenotype of Spastic paraplegia, intellectual disability, nystagmus, and obesity. Patient 125 with c.1807G > C in the *GNE* (NM_0011282272) showed facial dysmorphism, congenital heart defect, vertebral anomaly, diaphragmatic hernia, and skeletal dysplasia. GNE is responsible for two genetic diseases: GNE related myopathy and sialuria. c.1807G > C is not located on hot spot region for sialuria.^30^ Patient 132 with c.1304 G > A in the *LMNA* (NM_170707.3) showed failure to thrive, muscle weakness, and contractures at age 2 years. She showed spasticity, hyper Ig E level, skin rash, arthritis on follow‐up physical examination, which is not consistent with Muscular dystrophy, congenital. Patient 287 with c.7927‐1G > A in the *BPTF* gene (NM_182641.3) showed intrauterine growth restriction, global developmental delay, facial dysmorphism, facial asymmetry, clinodactyly, which was considered as Silver‐Russell syndrome. However, 11p15 methylation‐specific multiplex ligation‐dependent probe amplification analysis was normal. She did not have skeletal anomalies, such as, pes planus, broad halluces, and pes planus and ophthalmologic problems caused by *BPTF* variant.

**FIGURE 3:**
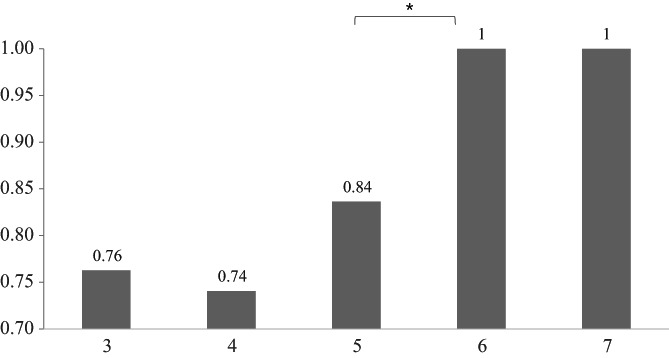
Distribution of symptom similarity scores of patient phenotypes and genetic phenotypes suggested by the automated system. ^*^ *P* < .05

The average numbers of HPO items in patients without an identified variant, in those with a confirmed variant, and in those with a rejected variant were 6.4 ± 5.0, 7.4 ± 5.3, and 9.0 ± 4.9, respectively (*P* > .05). There was no significant difference in the probability of confirmation of a certain variant identified by EVIDENCE among the affected organ types (Table S3). Notably, the confirmation rate was significantly higher when the similarity score of a gene variant was >5 than when it was <5 points (*P* = .032, Figure 3).

### 3.4 Genotypic and phenotypic diversity of enrolled patients

**FIGURE 4:**
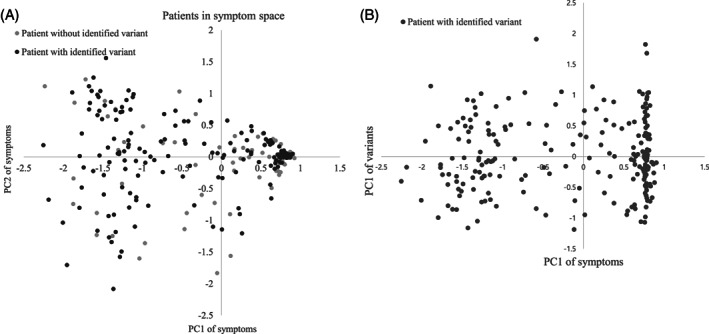
A, Distribution of patients in symptom space. B, Distribution of patients with identified variants in symptom and genetic variation space

No significant differences were observed in the distribution of clinical symptoms between patients with and without a variant identified by EVIDENCE (by 2‐dimensional Kolmogorov‐Smirnov test; Figure 4A). A similarity of principal component (PC1) values in symptom PCA of two patients implies a similarity in symptoms between these patients (Figure 4B).

Based on data shown in Figure 4B, we divided patients with identified variants into two groups using a PC1 of 0.5 in symptom PCA as a threshold. Of the 195 patients with identified variants, 89 (45.6%) were clustered together in PC1 of symptom PCA ranging from 0.5 to 0.93 (13% of total symptom PC1 range). In other words, the phenotypes of 45.6% patients covered only 13% of the total symptom PCA space, with the remaining 54.4% of patients covering the other 87%. The two patient groups showed similar diversity of genetic variants, as shown by the Student's *t* test of PC1 of genetic variation PCA (*P* = .899).

### 3.5 Identification of ultra‐rare genetic disorders and its impact on clinical management

An ultra‐rare genetic disease is defined as affecting 1 < 50 000 individuals.^31^ In this study, 91 (75.2%) of 121 genetic disorders identified were ultra‐rare genetic disease (Table S1).

Three patients changed their clinical management after WES and 82 patients received disease‐specific surveillance for the known complications. For example, patient 10 presented with hypernatremia at age 1 year, which did not respond to desmopressin. Thiazide was given with the clinical impression of nephrogenic diabetes insipidus, although no pathogenic variants were identified in *AVPR2* and *AQP2*. EVIDENCE suggested c.1679 T > C (p.Leu560Pro) and c.382C > T (p.Arg128Ter) in *SLC12A1*; subsequently, she was confirmed as having Bartter syndrome, type 1, and discontinued unnecessary medications. Patient 44 presented with recurrent renal stones after renal transplantation at the age of 41 years. The kidney biopsy showed accumulation of calcium oxalate crystals, but no pathogenic variant of *AGXT* was found. Homozygous *APRT* mutations, c.294G > A (p.Trp98Ter), were identified and adenine phosphoribosyltransferase deficiency was diagnosed. Thereafter, the patient was treated with allopurinol to inhibit renal stone progression. Patient 72 showed lactic acidosis and hyperbilirubinemia at birth, with a negative mitochondrial genome test. She had been on a low carbohydrate diet because mitochondrial disease with a respiratory chain defect was suspected, owing to high lactic acid level. However, after WES, she was diagnosed with pyruvate carboxylase deficiency caused by c.2874dup (p.Phe959ValfsTer8) and c.179dup (p.Ile61HisfsTer9) in *PC* and dietary management was changed to a high carbohydrate diet, which improved the biochemical findings of the patient.

## 4 DISCUSSION

EVIDENCE, an automated variant prioritization system, was found to be useful in the entire WES process, including raw data processing, variant prioritization, and measurement of phenotypic similarity between patients and suggested candidate diseases.

The diagnostic yield of EVIDENCE in the present study (42.7%) was comparable with that previously reported for automated systems (30‐35%).^9^, ^15^, ^16^ This finding was important, as the phenotypes of the enrolled patients were quite heterogeneous, broadly dispersed, and not limited to certain organ categories. In addition, most of the diseases identified (75.2%) were ultra‐rare genetic diseases. The distribution of inheritance patterns in the identified genetic disorders was generally similar to those in these reported studies, except that the proportion of autosomal dominant disorders was higher in our study.^6^, ^8^, ^10^

Diagnosis rates over 40% have been reported in the absence of an automated system in patients with select disease phenotypes, including hearing loss, visual impairment, or abnormalities of the musculoskeletal system, as well as in patients with critical conditions and in new‐borns presenting with symptoms.^4^, ^8^, ^32^ Moreover, in the absence of an automated system, a large amount of time is required to interpret a significant number of variants in each patient.^6^, ^11^ The results presented here indicate that our automated variant prioritization system can contribute to diagnosing various types of genetic diseases with comparable accuracy, but with much greater speed, than non‐automated analyses.

The comparable rate of diagnosis achieved by the automated system may be due to its high‐performance efficiency. Based on the systemic analysis of each variant and the relationship of each variant to patient phenotype, the results of this analysis suggested an average of 65 variants, putatively responsible for the phenotype of the patient. This reduction in variant number shortened the time required to select the variant most probably responsible for the phenotype of that patient, and it minimized the likelihood of missing the disease‐causing variant.

Another factor responsible for the high diagnostic rate of this automated system was that a substantial proportion of the variants suggested by the system were VUSs. These VUSs were subsequently tested in family member segregation analysis and phenotype reassessment, as it is unclear whether VUSs are causative variants in the absence of segregation analysis and clinical reassessment. Updated information on variants in genome databases can result in VUSs being classified as pathogenic or benign.^12^, ^33^, ^34^ Before family member testing and clinical reassessment, 67.3% of the patients in our study had variants classified as pathogenic or probably pathogenic. After family member testing and reassessment; however, 97.2% of our patients had these variants. Following family member testing and clinical reassessment, 59 (90.8%) of 65 VUSs were reclassified as pathogenic or probably pathogenic. Two of 6 VUS variants was compound heterozygous with other pathogenic and probably pathogenic variants, and highly matched with disease relevant symptoms. One patient had maternal UPD 9 encompassing region located on VUS variant. Thus, these 3 patients were performed a definite diagnosis. The remaining 3 patients (Patient 20, 89, and 124) with VUS also showed highly specific phenotypes associated with each variant. Among two of 3 patients performing family member testing, patient 124 had symptomatic sibling with the same variant and patient 89 was confirmed in trans phase as a homozygous state. Patient 20 did not perform family member testing. Thus, these 3 variants were considered as responsible for patients' phenotypes. Therefore, all these 6 VUS were included in the diagnostic yield.

In variant prioritizing systems, the score of the top‐ranked variant increases when patient symptoms more precisely match those caused by the responsible gene, and when the number of HPO terms of a patient increases.^35^ The present study found no significant differences in the average numbers of HPO terms and organ types between patients in whom causative variants have and have not been identified. This finding was probably related to the wide range of phenotypes among our patients. In contrast, similarity scores were calculated as previously described^27^, ^28^ with slight modifications. Maximal depth of the common ancestor node of two symptoms in the HPO tree structure was used instead of its information content because the latter depends on symptom‐disease mapping data. Notably, we observed that scores ≥5 points were associated with a significantly higher probability of confirmation of a certain variant as causative. The ancestor HPO term has relatively low accuracy; however, improvements in the determination of similarity scores and a more detailed description of symptoms are required to enhance the accuracy of variant prioritizing systems.

Most importantly, WES using EVIDENCE exerted a significant impact on the clinical management of diagnosed patients. Previous studies have reported that WES changed clinical managements in 5% to 65% of diagnosed patients.^8^, ^32^, ^36^ In our study, EVIDENCE helped to change clinical management in about half of the diagnosed patients, and this change was critical in some cases for the improvement of patient care, for medication or dietary management, or for disease‐specific surveillance.

This study had several limitations. First, most of the patients were pediatric patients. Pediatric patients have a higher likelihood of genetic diseases than do adults, which may have contributed to the relatively high rate of diagnosis in our patient cohort. Second, family member testing could not be performed in 46 of the 141 patients diagnosed with genetic diseases because samples from family members were unavailable. Third, the use of the ClinVar and Uniprot databases may change variant classification as the variant information is updated, and even two databases are not consistent, given the concordance rate of approximately 88%.^37^ Therefore, the rule application can be added or removed, according to the updated information, and the variant classification can be changed. Fourth, the actual causative variant may have been missed by the automated system.

In conclusion, the rate of detection of variants by the automated system did not differ significantly in patients who did and did not undergo genetic testing before WES. This automated system achieved a comparable diagnostic yield in patients with a broad range of genetic diseases, suggesting that WES may be one of the first diagnostic methods used in patients suspected of having a genetic disease, and that the automated system can facilitate the diagnostic process. This new method is available to others (https://portal.3billion.io/) allowing the efficiency of this system to be evaluated by other groups for larger patient cohorts. Phenotype‐centric tools, such as, Phenovar or Exomiser have been recently developed.^9^, ^15^, ^16^ EVIDENCE also uses a phenotype‐centric approach, but prioritized variants are ranked in order in EVIDENCE (but not in the other systems). Furthermore, we are currently developing an ungraded system that prioritizes variants by merging the variant classification and similarity score into a single system, which will improve the analytical methods used to evaluate variants by EVIDENCE.

## CONFLICTS OF INTEREST

The authors declare no conflict of interest.

## AUTHOR CONTRIBUTIONS

BHL designed the study. GHS, TK, IHC, and BHL drafted the manuscript and analyzed the data. GHS, AO, YL, IHC, JC, HL, HGK, HYC, MHC, YJK, YHY, BE, and BHL treated the patients and performed all clinical analyses. GHS, SK, JL, DW, and CK developed EVIDENCE. GHS, JYP, and TK performed genetic analyses and interpreted variant data. RJD critically revised the manuscript. All authors were involved in analyzing and interpreting data. All authors have read and approved the final manuscript.

### PEER REVIEW

The peer review history for this article is available at https://publons.com/publon/10.1111/cge.13848.

## Supporting information


**Supplementary Figure 1** A diagram highlighting each step of the filtering process used for the variants obtained from whole exome sequencing. The whole exome data included approximately 109 288 variants per patient. We first excluded variants with a high 5% minor allele frequency, which eliminated nearly 98% of the variants. This left approximately 10 702 variants. After the genes were matched to diseases, approximately 2756 variants remained. Finally, approximately 65 disease‐variant pairs remained to be manually curated after excluding the variants with a low impact, including probably benign, benign, and non‐coding variants with low evidence according to the ACMG guidelines and filtering based on the inheritance pattern.

**Supplementary Figure 2** Distribution of Human Phenotype Ontology (HPO) terms and patient symptoms in 330 patients (green dots: 16000 HPO terms; red dots: patient symptoms).

**Supplementary Table 1** Database information for variant annotation
**Supplementary Table 2.** Detailed information about the 167 confirmed variants in 141 patients.
**Supplementary Table 3.** Confirmation rate according to the type of involved organ in patients with confirmed and rejected variant.

**Supplementary File 1** Description of similarity score formula

**Appendix S1** Supporting Information.

## Data Availability

The variants reported in this study are available from supplementary table.
