# Supplementary figures and images for: Diagnostic yield and clinical utility of whole exome sequencing using an automated variant prioritization system, EVIDENCE
Source: Clin Genet. 2020 Sep 17;98(6):562–70. doi: 10.1111/cge.13848 (PMC7756481; doi:10.1111/cge.13848)

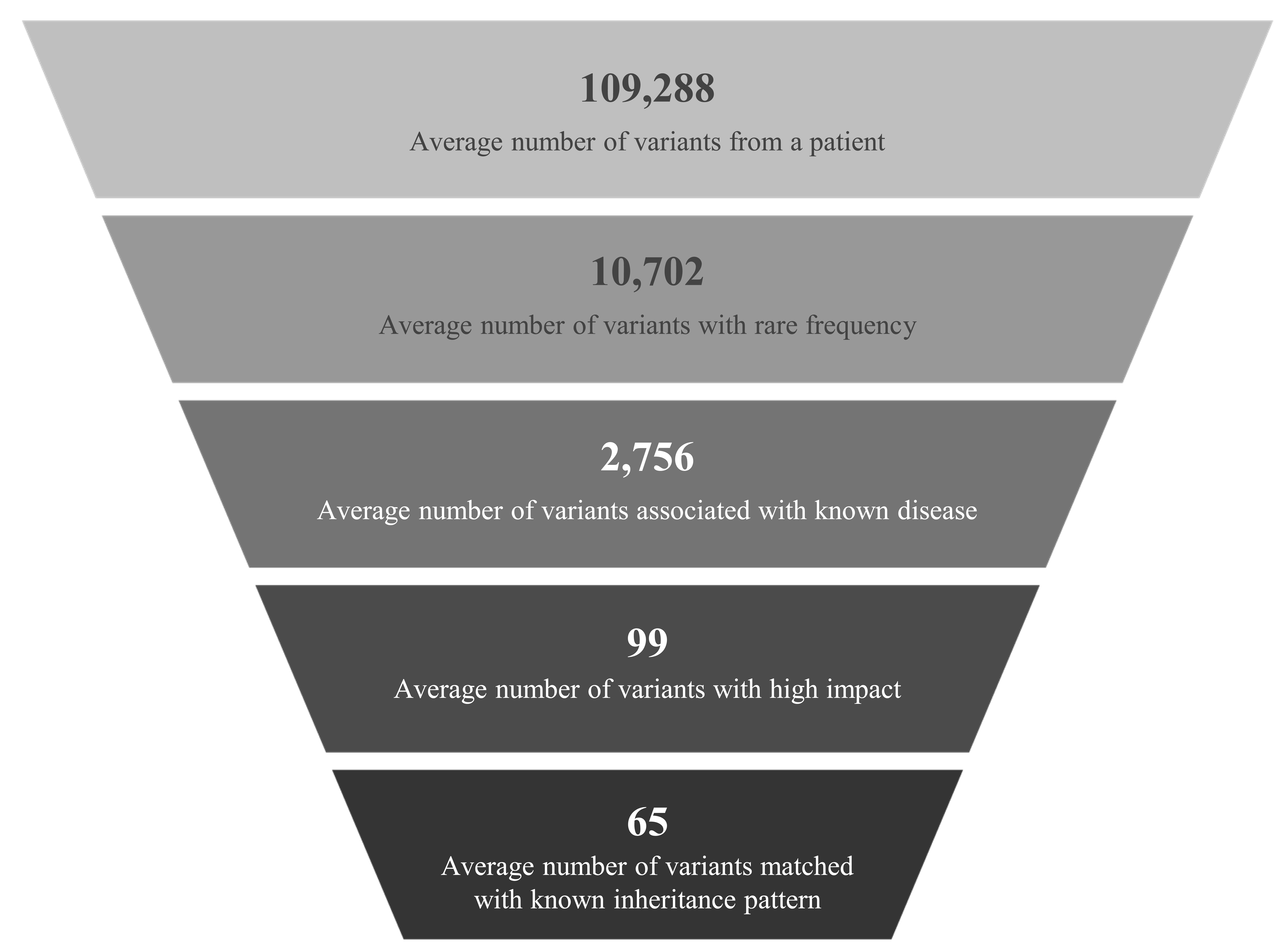

Supplement: Supplementary file 1 — Supplementary Figure 1 A diagram highlighting each step of the filtering process used for the variants obtained from whole exome sequencing. The whole exome data included approximately 109 288 variants per patient. We first excluded variants with a high 5% minor allele frequency, which eliminated nearly 98% of the variants. This left approximately 10 702 variants. After the genes were matched to diseases, approximately 2756 variants remained. Finally, approximately 65 disease‐variant pairs remained to be manually curated after excluding the variants with a low impact, including probably benign, benign, and non‐coding variants with low evidence according to the ACMG guidelines and filtering based on the inheritance pattern. [file CGE-98-562-s001.tif]

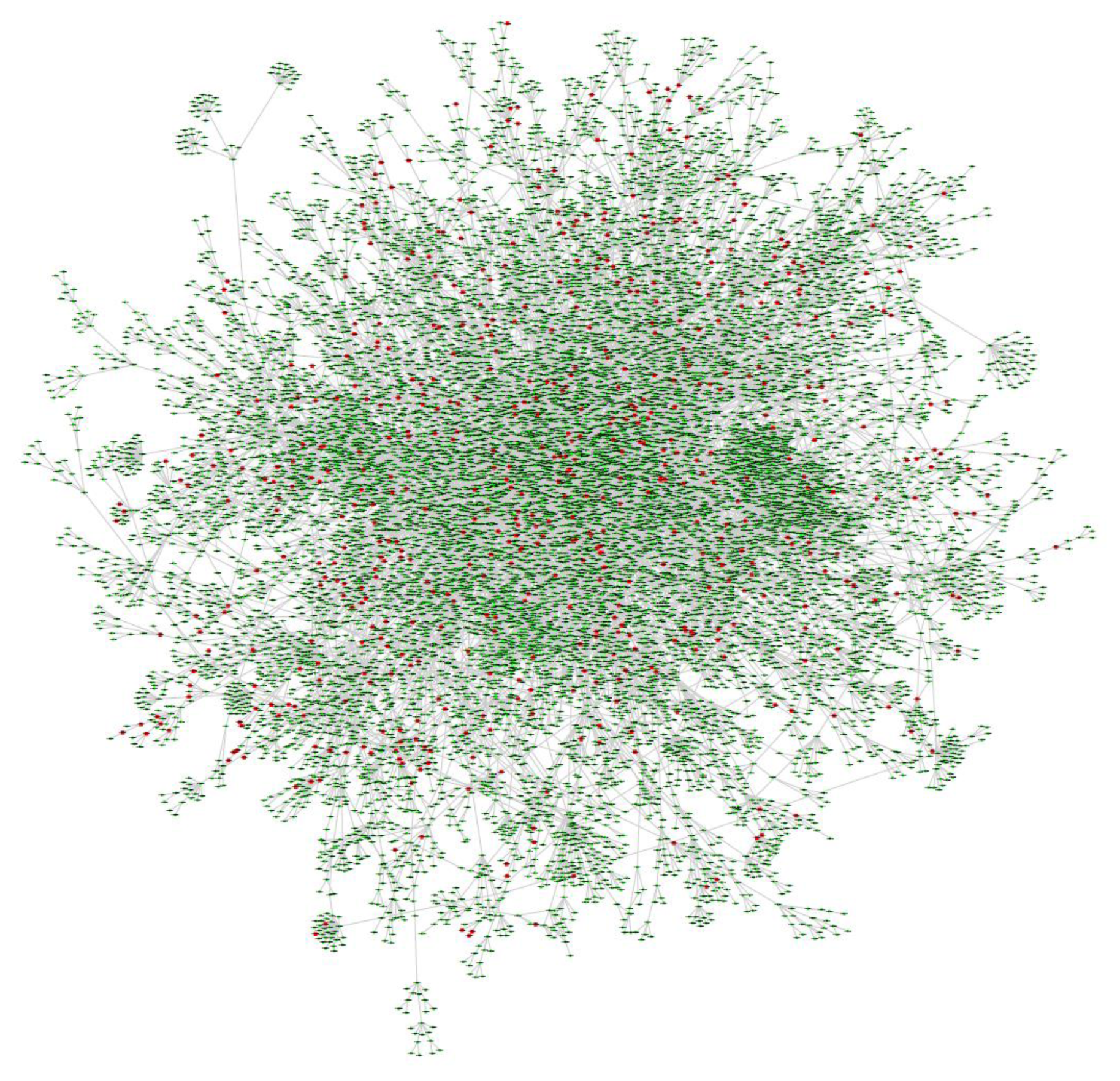

Supplement: Supplementary file 2 — Supplementary Figure 2 Distribution of Human Phenotype Ontology (HPO) terms and patient symptoms in 330 patients (green dots: 16000 HPO terms; red dots: patient symptoms). [file CGE-98-562-s002.tif]
