# Supplementary material for: Diagnostic yield and clinical utility of whole exome sequencing using an automated variant prioritization system, EVIDENCE
Source: Clin Genet. 2020 Sep 17;98(6):562–70. doi: 10.1111/cge.13848 (PMC7756481; doi:10.1111/cge.13848)
Supplement: Supplementary file 4 — Supplementary File 1 Description of similarity score formula [file CGE-98-562-s004.docx]

Supplementary file 1. Description of similarity score formula

Similarity score formula:

$$w\left( p,\mathbb{S} \right)=max\{s\in S:MCA_{d}\left( p,s \right)\} (1)$$

$$d(\mathbb{S}_{p},\mathbb{S}_{d}) = \frac{1}{|\mathbb{S}_{p}|}\sum_{s\in\mathbb{S}_{p}} w\left( p,\mathbb{S}_{p} \right) (2)$$

$$score\left( \mathbb{S}_{p},\mathbb{S}_{d} \right)=\frac{1}{2}\left( d\left( \mathbb{S}_{p},\mathbb{S}_{d} \right)+d\left( \mathbb{S}_{d},\mathbb{S}_{p} \right) \right) (3)$$

As in (1), weight w(p,S) of a symptom p to a set S of symptoms is the Maximum depth of Common Ancestors (MCA) of p and symptoms in S in HPO graph structure from HP:0000118 Phenotype abnormality. With this definition of weight, disease score of a set S_p_ of symptoms to other symptom set S_d_ (2) is defined as a sum of weights of p to S_d_ for all p in Sp divided by the number of symptoms in S_p_. It is more intutive for score (distance) to have the same value for score (A,B) and score (B,A) with different two elements A and B, which is called commutative. To make disease score be commutative, the final score is defined by d (S_p_, S_d_) + d(S_d_,Sp) divided by 2 as in (3)
