# Supplementary material for: Diagnostic yield and clinical utility of whole exome sequencing using an automated variant prioritization system, EVIDENCE
Source: Clin Genet. 2020 Sep 17;98(6):562–70. doi: 10.1111/cge.13848 (PMC7756481; doi:10.1111/cge.13848)

Reviewer’s uploaded VCF


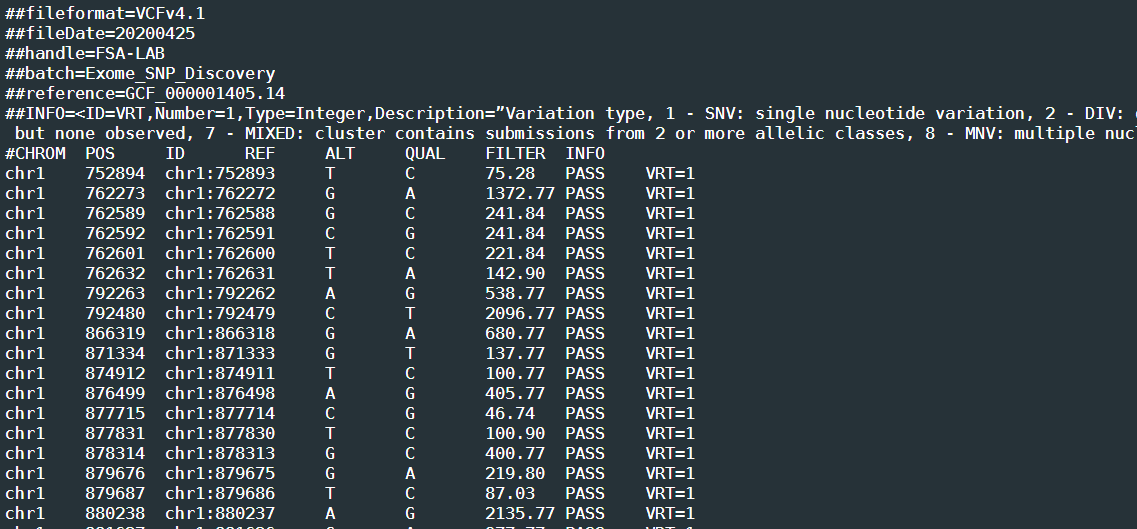


NA12878 VCF

<https://3billion-rawdata.s3.ap-northeast-2.amazonaws.com/sample/sample.vcf>


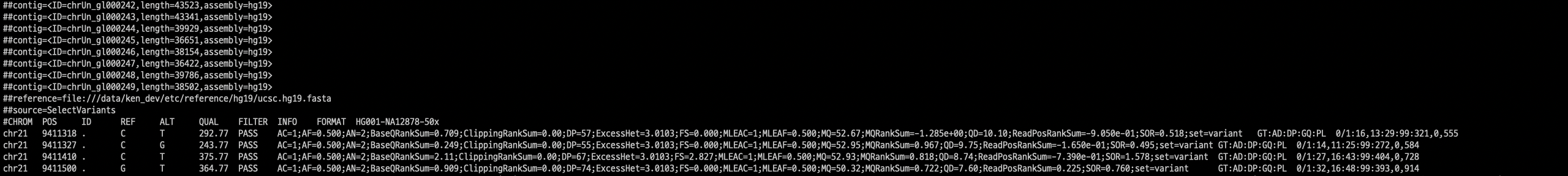


VCF Specification

<https://samtools.github.io/hts-specs/VCFv4.2.pdf>


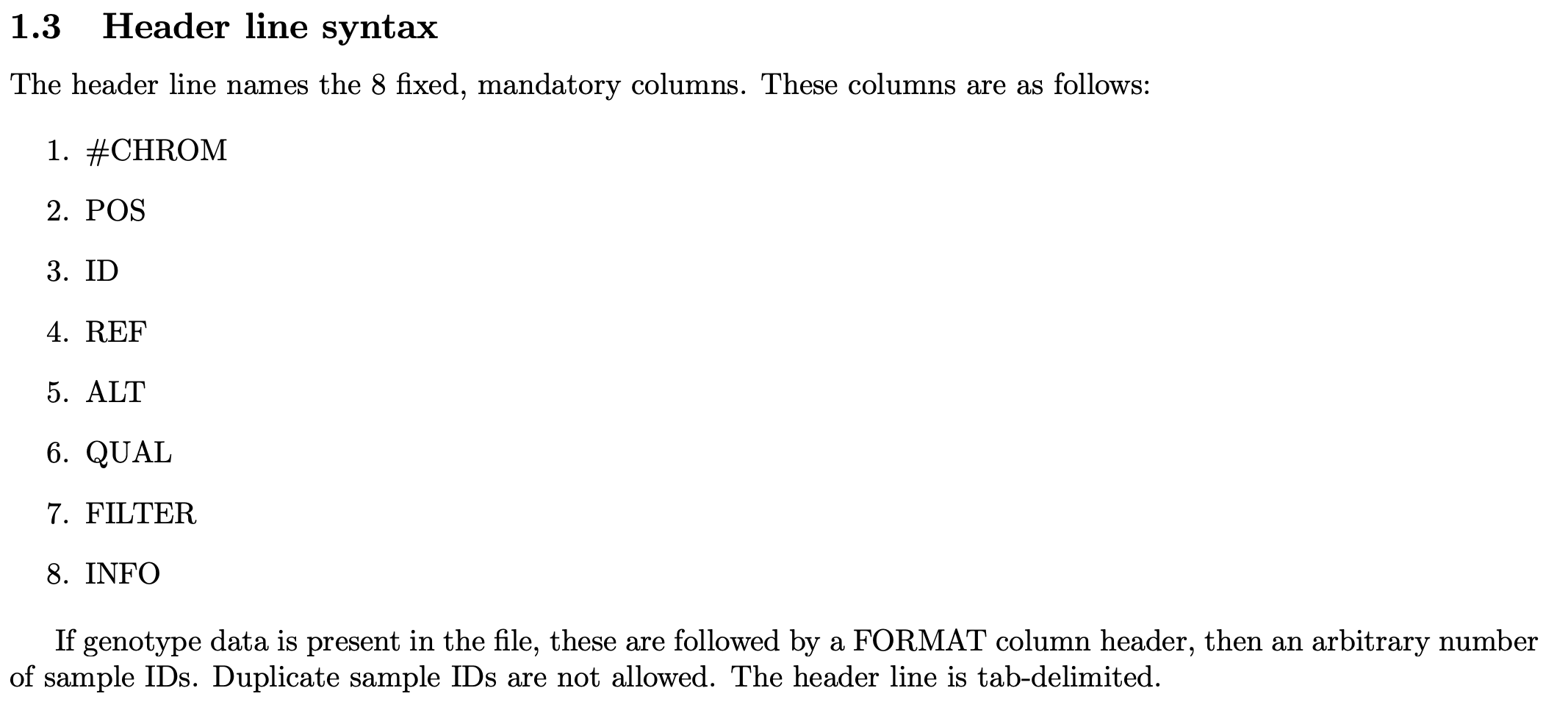

Supplement: Supplementary file 5 — Appendix S1 Supporting Information. [file CGE-98-562-s005.docx]
